# Longitudinal assessment of diffusion-weighted imaging during magnetic resonance-guided radiotherapy in head and neck cancer

**DOI:** 10.1186/s13014-025-02589-9

**Published:** 2025-01-29

**Authors:** Simon Boeke, Jonas Habrich, Sarah Kübler, Jessica Boldt, Fritz Schick, Konstantin Nikolaou, Jens Kübler, Cihan Gani, Maximilian Niyazi, Daniel Zips, Daniela Thorwarth

**Affiliations:** 1 Department of Radiation Oncology, University Hospital Tübingen, Tübingen, Germany; 2 German Cancer Consortium (DKTK), partner site Tübingen, and German Cancer Research Center (DKFZ), Heidelberg, Germany; 3 Section for Biomedical Physics, Department of Radiation Oncology, University Hospital Tübingen, Tübingen, Germany; 4 Section for Experimental Radiology, Department of Diagnostic and Interventional Radiology, University Hospital Tübingen, Tübingen, Germany; 5 Department of Diagnostic and Interventional Radiology, University Hospital Tübingen, Tübingen, Germany; 6 Department of Radiation Oncology, LMU University Hospital, LMU Munich, Munich, Germany; 7 Charité Clinic for Radiation Oncology and Radiation Therapy – University Medicine Berlin, Berlin, Germany

**Keywords:** MR-guided radiotherapy, Apparent diffusion coefficient, Quantitative magnetic resonance imaging, Head and neck cancer

## Abstract

**Background:**

For radiotherapy of head and neck cancer (HNC) magnetic resonance imaging (MRI) plays a pivotal role due to its high soft tissue contrast. Moreover, it offers the potential to acquire functional information through diffusion weighted imaging (DWI) with the potential to personalize treatment. The aim of this study was to acquire repetitive DWI during the course of online adaptive radiotherapy on an 1.5 T MR-linear accelerator (MR-Linac) for HNC patients and to investigate temporal changes of apparent diffusion coefficient (ADC) values of the tumor and subvolume levels.

**Methods:**

27 patients treated with curative RT on the 1.5 T MR-Linac with at least weekly DWI in treatment position were included into this prospective analysis and divided in four risk groups (HPV-status and localisation). Tumor and lymph node volumes (GTV-P/GTV-N) were delineated on b = 500 s/mm^2^ images while ADC maps were calculated using b = 150/200 and 500 s/mm^2^ images. Absolute and relative temporal changes of mean ADC values, tumor volumes and a high-risk subvolume (HRS) defined by low ADC tumor voxels (600 < ADC < 900 × 10^−6^ mm^2^/s) were analyzed. Relative changes of mean ADC values, tumor volumes and HRS were statistically tested using Wilcoxon-signed-rank test.

**Results:**

Median pretreatment ADC value for all patients resulted in 1167 × 10^−6^ mm^2^/s for GTV-P and 1002 × 10^−6^ mm^2^/s for GTV-N while absolute pretreatment tumor volume yielded 9.1 cm^3^ for GTV-P and 6.0 cm^3^ for GTV-N, respectively. Pretreatment HRS volumes were 1.5 cm^3^ for GTV-P and 1.3 cm^3^ for GTV-P and GTV-N. Median ADC values increase during 35 fractions of RT was 49% for GTV-P and 24% for GTV-N during RT. Median tumor volume decrease was 68% and 52% for GTV-P and GTV-N with a median HRS decrease of 93% and 87%. Significant differences from 0 for mean ADC were observed starting from week 1, for tumor volumes from week 2 for GTV-P and week 1 for GTV-N and for HRS in week 1 for GTV-P and week 2 for GTV-N.

**Conclusion:**

Longitudinal DWI acquisition in HNC is feasible on a MR-Linac during the course of online adaptive MR-guided radiotherapy. Changes in ADC and volumes can be assessed, but future work needs to explore the potential for biologically guided treatment individualization.

*Trial registration:* NCT04172753, actual study start: 09.05.2018.

## Background

Radiotherapy (RT) is one of the cornerstones in the treatment of head and neck cancer (HNC). Different prognostic factors exist for HNC, with the most important being human papilloma virus 16 (HPV) and smoking status in oropharyngeal cancers (OPC) and mainly tumor stage for other tumor sites [1–3]. In depth research has been done on imaging biomarkers in HNC, with partially contradictory results. Positron emission tomography either with FDG or hypoxia specific tracers were described to offer prognostic potential and have been used to escalate or de-escalate treatment by dose painting [4–8].

Due to its superior soft tissue contrast magnetic resonance imaging (MRI) plays a pivotal role in target volume definition and might prove beneficial for automation of these time comsuming steps in HNC [9–11]. Besides the anatomical information from MRI, it offers the possibility to acquire functional imaging such as dynamic contrast enhanced (DCE) and diffusion-weighted imaging (DWI). Several single center studies looked at different imaging biomarkers with regards to prognostic information and technical validation with contradicting results, especially in HPV associated OPC [12–17]. Lambrecht et al. showed prognostic information of DWI acquired before radiotherapy with regards to local control [18]. Contradictory to these results, there are observations in HPV associated OPC, that DWI is not prognostic and may not be compared to non-HPV associated HNC [15, 19].

Due to the complexity and limited availability of MRI, only a few studies were reported focusing on longitudinal imaging during the course of fractionated RT [17, 20–22]. In recent years the technique of magnetic resonance linear accelerators (MR-Linac) has been introduced and first data were published for MR-guided RT for HNC [23, 24]. Initial results showed the feasibility of MR-guided RT for HNC, providing the potential to acquire longitudinal anatomical imaging with high resolution and functional imaging during the course of fractionated radiotherapy [23–25]. Moreover, in our department a potential quantitative imaging biomarker (QIB) based on a cluster of ADC values has been established in a preclinical model, suggesting a high-risk subvolume (HRS), which could be used for individualized dose prescription [26]. A validation on a clinical cohort of patients has been performed in which the retrained HRS was found to be significantly associated to outcome after primary radiochemotherapy, so this ADC-based HRS might serve as a biomarker for stratification or therapy individualization of HNC patients [27].

The aim of the present study was to explore the feasibility of online acquired, longitudinal DWI with an exploratory analysis of changes in different DWI parameters during the course of MR-guided RT in HNC patients.

## Methods

### Patient and treatment characteristics

A total of n = 28 patients with HNC, which were treated on the 1.5 T MR-Linac (Unity, Elekta AB, Sweden) between October 2018 and December 2021 have been prospectively included into this analysis within a enclosed project funded by the German Research Council. All patients gave written informed consent to be treated within this prospective trial of MR-guided adaptive RT which was approved by the local ethics committee (no. 659/2017BO1, NCT04172753). All patients were immobilized with a 5-point radiotherapy mask on a dedicated head step for the use on the 1.5 T MR-Linac in a neutral neck position [13]. The planning-CT of 2 to 3 mm slice thickness and all MRIs have been acquired in this setup as shown in detail elsewhere [13, 14, 23, 24]. Patients were instructed to limit swallowing whenever possible. Treatment consisted of fractionated RT with 60 Gy and 54 Gy in 30 fractions to the macroscopic tumor, the high risk area and the elective volume with a sequential boost to the macroscopic tumor of 10 Gy in 5 fractions, consistent with international guidelines. Concomitant weekly Cisplatin was administered. The treatment was performed on the MR-Linac with an online “adapt-to-position” workflow and offline adaptation in case of large anatomical deviations based on the treating physicians discretion [24]. Detailed patient characteristics are shown in Table 1. Follow-up was according to clinical routine with a FDG-PET/CT after 3 months in case of nodal involvement and further clinical and radiological examinations every 3 months. For OPC, risk groups have been defined according to Ang et al. with stratification based on p16-, smoking-status and TNM stage [1]. The remaining six non-OPC patients have been included into a fourth patient group.

**Table 1:** Patient characteristics

Patient characteristics		N = 27
Age (median, range)		67 (39–78)
Primary tumor site	Hypopharyngeal Oropharyngeal Supraglottic larynx	5 21 1
Primary tumor (T) stage	T1 T2 T3 T4	0 9 13 5
Nodal tumor (N) stage	N0 N1 N2 N2a N2b N2c	0 9* 8^+^ 0 5 4
p16-status	Positive Negative	15^#^ 12
Smoking status	Current Former Never	9 9 9

^*^n = 6 p16 positive

^+^n = 8 p16 positive

^#^n = 1 non-OPC

### Imaging protocol

Imaging of all patients was performed on the 1.5 T MR-Linac except for pretreatment images of patient 25 which were acquired on a 1.5 T diagnostic scanner (Ingenia, Philips). The protocol consisted of a single-shot echo-planar imaging (SS-EPI) DWI as well as T1- and/or T2-weighted anatomical imaging. For the first eleven patients the acquired b-values of the DWI sequence were 0, 200, 500 and 800 s/mm^2^ while starting with patient 12, the DWI sequence was adapted according to the recommendations of the MR-Linac consortium [28] and b-values 0, 150 and 500 s/mm^2^ were acquired. For patient 2 the b-value 500 s/mm^2^ was not part of the DWI sequence pretreatment and in fraction four of RT, while patient one had to be excluded from analysis due to missing DWI data. Details about the sequence parameters of both DWI as well as the two anatomical MRI protocols are provided in Table 2. Patients were imaged in RT position either directly before the start, during beam on or after completion of RT on the MR-Linac. The imaging protocol was applied during simulation before the start of radiotherapy and sequentially during RT, approximately once per week.

**Table 2:** Details of the sequence parameters

Parameter	EPI3b	EPI4b	T1w MRI (T1_3D_Tra)	T2w MRI (T2_3D_Tra)
Sequence type	SS-SE-EPI^a^	SS-SE-EPI	FFE^b^ 3D	MS-SE-TSE^c^ 3D
Acquisition voxel size (mm^3^)	3 × 3 × 4	3 × 3 × 4.8	1.2 × 1.2 × 2.4	1.2 × 1.2 × 2.2
Slice gap (mm)	0	0	-1.2	-1.1
Field of view (mm^3^)	400 × 400 × 100	400 × 400 × 202	520 × 298 × 250	520 × 298 × 250
TR/TE (ms)	4811/68	10,392/107	13/4.5	2100/375
Flip angle (°)	90–180	90–180	27	90–(180)
TSE factor	–	–	–	150
Water-fat shift (pix)/bandwidth (Hz)	11.197/19.4	11.180/19.4	0.653/332.6	0.473/459.3
b-values (s/mm^2^) (averages)	0 (3), 150 (5), 500 (8)	0 (2), 200 (3), 500 (4), 800 (6)	–	–
Fat suppression	SPAIR^d^	SPAIR^d^	No	No
Duration (min)	3:32	7:17	5:48	6:03

^a^Single-shot spin-echo echo-planar imaging

^b^Fast Field Echo

^c^Multi-shot spin-echo turbo spin-echo

^d^Spectral Attenuated Inversion Recovery

### Image processing

Volumes of interest (VOIs) defined as primary tumors (GTV-P) and all conspicuous lymph nodes (GTV-N) were delineated by a board-certified radiation oncologist (SB) on the b-500 images with the open-source software 3D Slicer (Version 4.10) taking the anatomical images as visual input to ensure the capture of the whole tumor volume. Then, ADC maps were calculated using an in-house written python script (version 3.8.10) and b-values 150/200 and 500 s/mm.^2^ with the mono-exponential model [29]

$${\text{SI}} = {\text{SI}}_{0} \cdot {\text{e}}^{{ - {\text{b}} \cdot {\text{ADC}}}} .$$

Here SI is defined as the signal intensity and SI_0_ as the calculated signal intensity at b-value 0 s/mm^2^.

Furthermore, a high-risk subvolume (HRS) was defined, representing potentially radioresistant tumor subregions [26] inside both GTV-P and GTV-N, separately. This HRS was defined as all voxels inside the GTV-P or GTV-N with ADC values in the range of 600–900 × 10^−6^ mm^2^/s. ADC thresholds were used from a prior investigation [27] and adapted for the use on the MR-Linac to the known underestimation of ADC on the MR-Linac [13, 30, 31].

### Statistics

GTV-P and GTV-N volumes as well as mean ADC values (ADC_mean_) and HRS volume were calculated using Matlab 2020a (MathWorks, Natick, MA, USA). Target volumes smaller than 1 cm^3^ and HRS smaller than 0.2 cm^3^ were excluded from further analysis. Absolute values of ADC and volumes were evaluated for all patients for baseline and every week of radiotherapy. Furthermore, linear regression as a function of treatment time regarding mean ADC values as well as volumes of GTV-P, GVT-N and HRS was fitted for all risk groups with Matlab. Additionally, relative changes of ADC and volume from baseline to every week of RT were evaluated for every risk group. One-sample Wilcoxon-signed-rank test with a significance level of 5% was used to evaluate relative changes of ADC_mean_, absolute tumor volumes and HRS volumes in every week of radiotherapy for a significant difference from 0 using SPSS statistical package 28.0.0.0 (SPSS Inc, Chicago, Illinois).

## Results

**Fig. 1:**
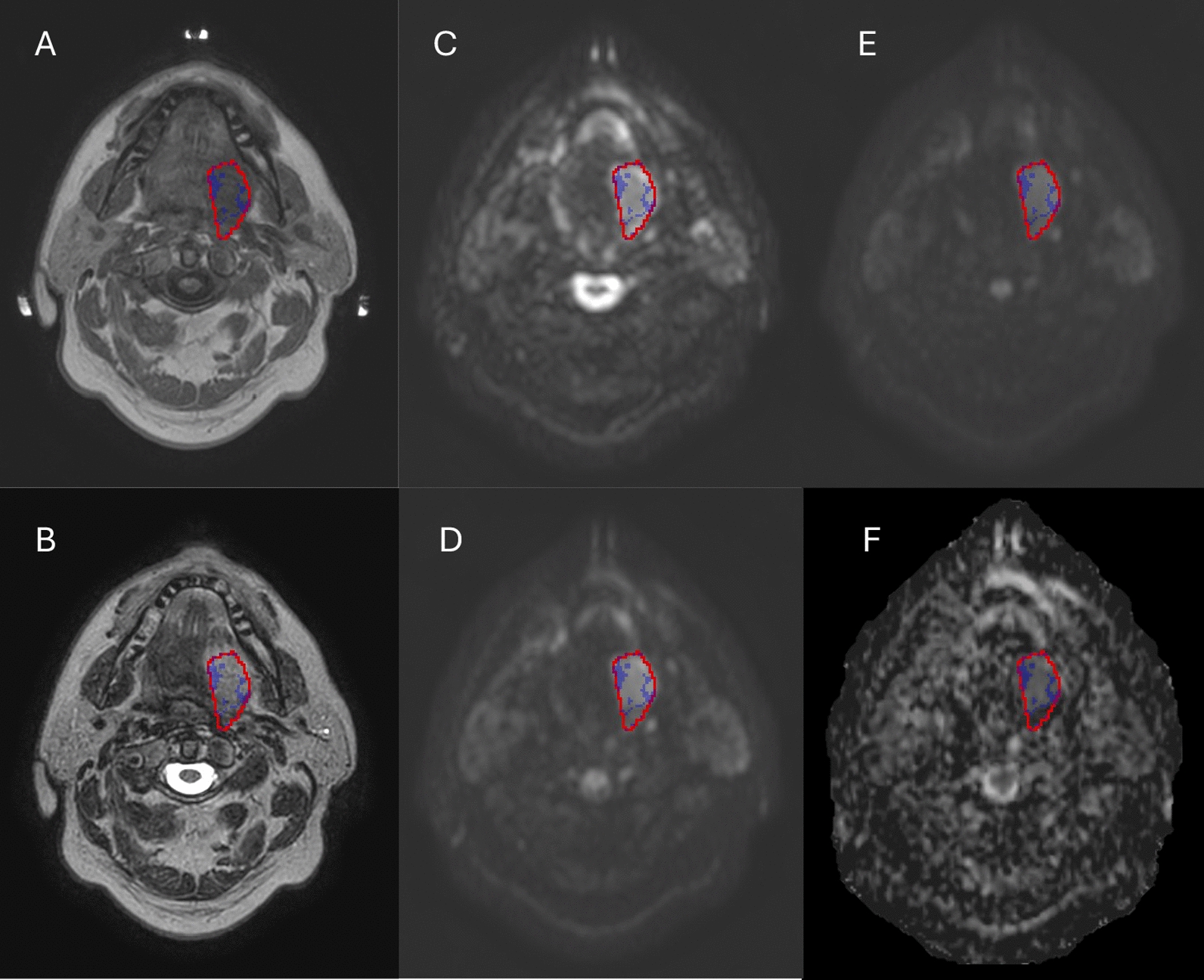
Exemplary pretreatment images of patient #17 including T1w (**A**), T2w (**B**), b0 image (**C**), b150 image (**D**), b500 image (**E**) and calculated ADC map (**F**) with GTV depicted in red and high-risk subvolume (600 < ADC < 900 × 10^−6^ mm^2^/s) in blue

A total of 28 patients have been treated on the MR-Linac out of which 27 primary lesions as well as 39 lymph nodes from 27 patients were available for analysis. Median (interquartile range) number of scans per patient during treatment was 7 (1). Exemplary pretreatment anatomical as well as DW images for patient 17 including delineations of the primary tumor and calculated HRS are shown in Fig. 1. At the time of analysis median follow up was 40.4 months, with five patients having died due to metastatic disease and one likely due to pneumonia after aspiration. One patient with a combined hypopharyngeal and proximal esophageal carcinoma (in initial endoscopy distant to each other) showed a recurrence in the proximal esophagus 18 months after completion of the treatment with no recurrence in the hypopharynx. No isolated local or regional recurrence have been observed in the cohort.

**Fig. 2:**
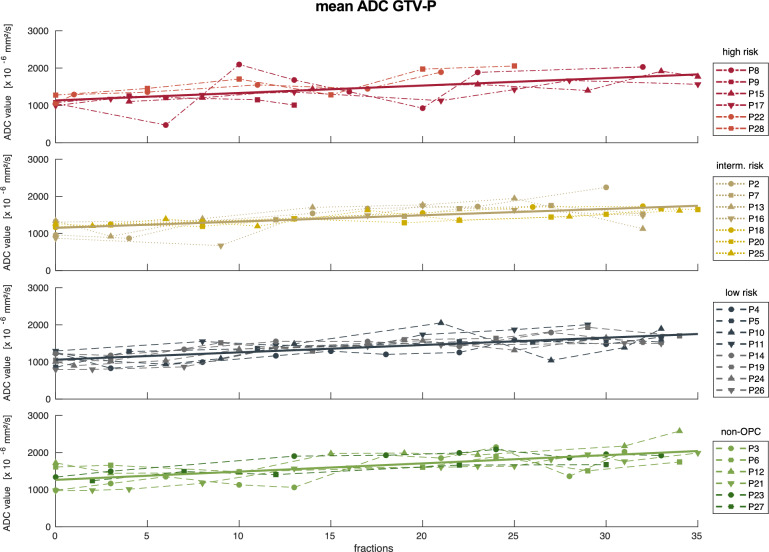
Mean ADC values for GTV-P over the course of radiotherapy for high (red), intermediate (gold), low risk (blue) and non-oropharyngeal (green) patients (according to Ang et al. [1]. Solid lines represent linear regression for each group of patients

**Fig. 3:**
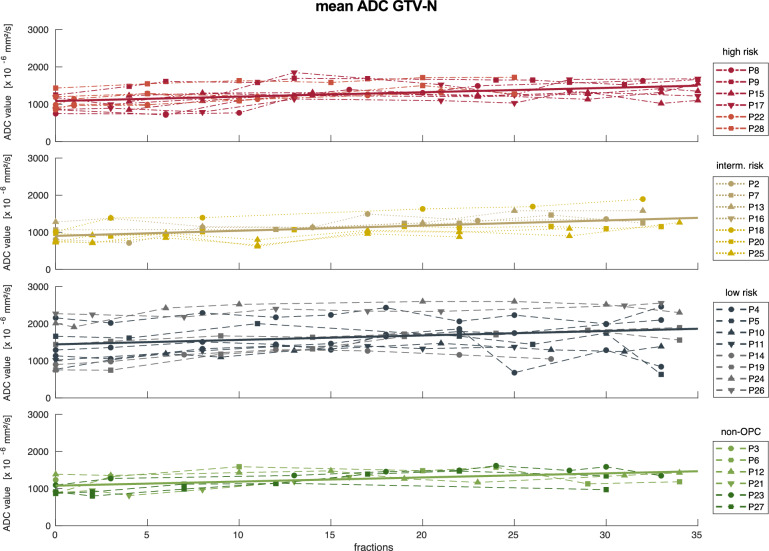
Mean ADC values for GTV-N over the course of radiotherapy for high (red), intermediate (gold), low risk (blue) and non-oropharyngeal (green) patients (according to Ang et al. [1]. Solid lines represent linear regression for each group of patients

Median (interquartile range) pretreatment ADC value for all patients amounted to 1167 (282) × 10^−6^ mm^2^/s for GTV-P and 1002 (398) × 10^−6^ mm^2^/s for GTV-N, respectively. Over the course of radiotherapy, the ADC_mean_ of primary lesions increased to 1687 (381) × 10^−6^ mm^2^/s and 1420 (424) × 10^−6^ mm^2^/s of conspicuous lymph nodes in the last week of radiotherapy. The temporal changes including linear regression of ADC_mean_ for GTV-P and GTV-N over the course of radiotherapy depending on the patient’s risk classification are displayed in Figs. 2 and 3 for all patients.

**Fig. 4:**
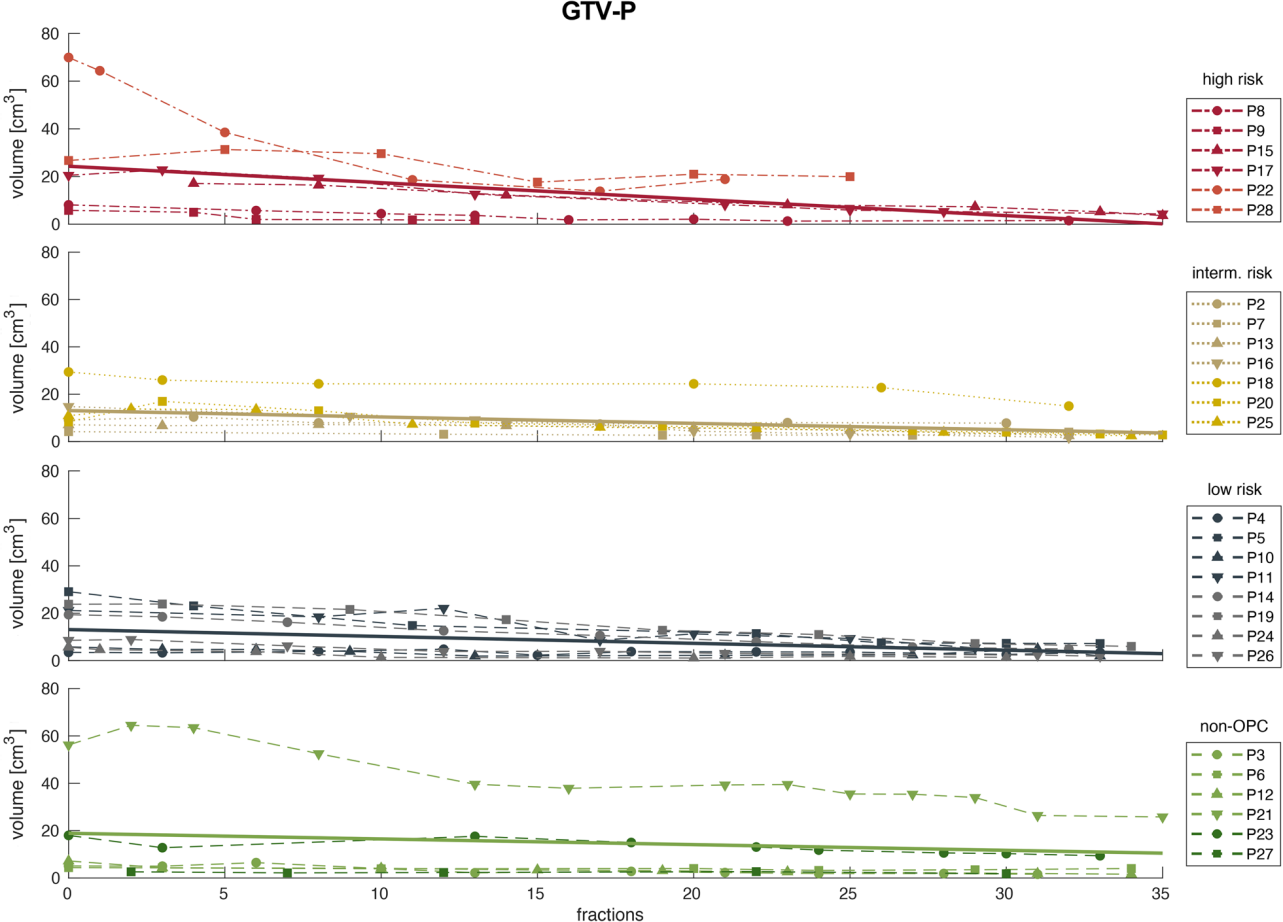
Volumes of GTV-P over the course of radiotherapy for high (red), intermediate (gold), low risk (blue) and non-oropharyngeal (green) patients. Solid lines represent linear regression for each group of patients

**Fig. 5:**
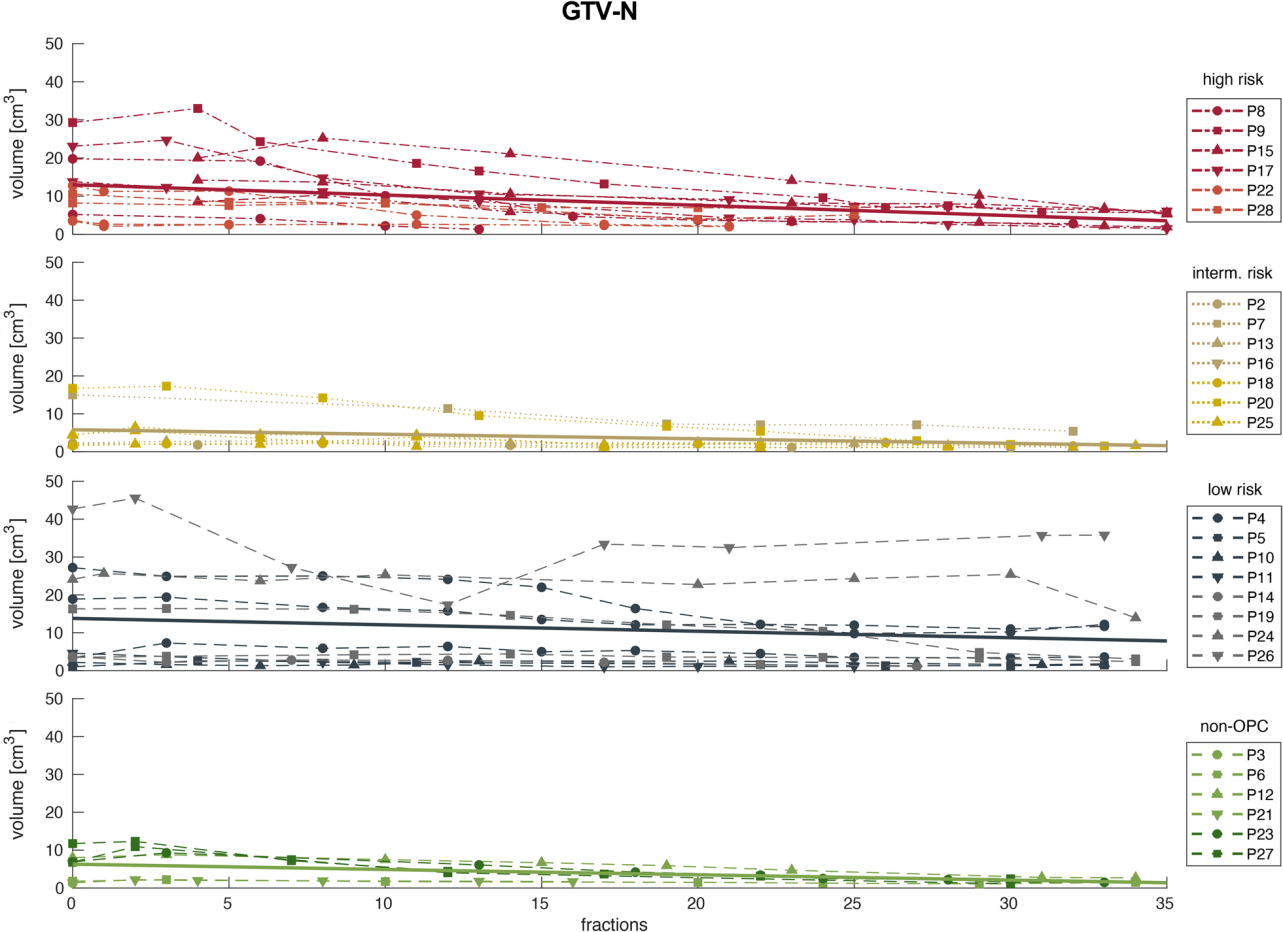
Volumes of GTV-N over the course of radiotherapy for high (red), intermediate (gold), low risk (blue) and non-oropharyngeal (green) patients. Solid lines represent linear regression for each group of patients

Regarding tumor volumes, median pretreatment GTV-P volume was 9.1 (15.4) cm^3^ while GTV-N displayed a median size of 6.0 (12.2) cm^3^. Median primary tumor volumes decreased over the course of radiotherapy to 3.6 (3.1) cm^3^ in the last week of RT. In contrast, the conspicuous lymph nodes had a median volume of 2.8 (4.4) cm^3^ in the last week of RT. For all patients, trends of absolute tumor volumes and linear regression are visualized in Figs. 4 and 5 for GTV-P and GTV-N, respectively.

**Fig. 6:**
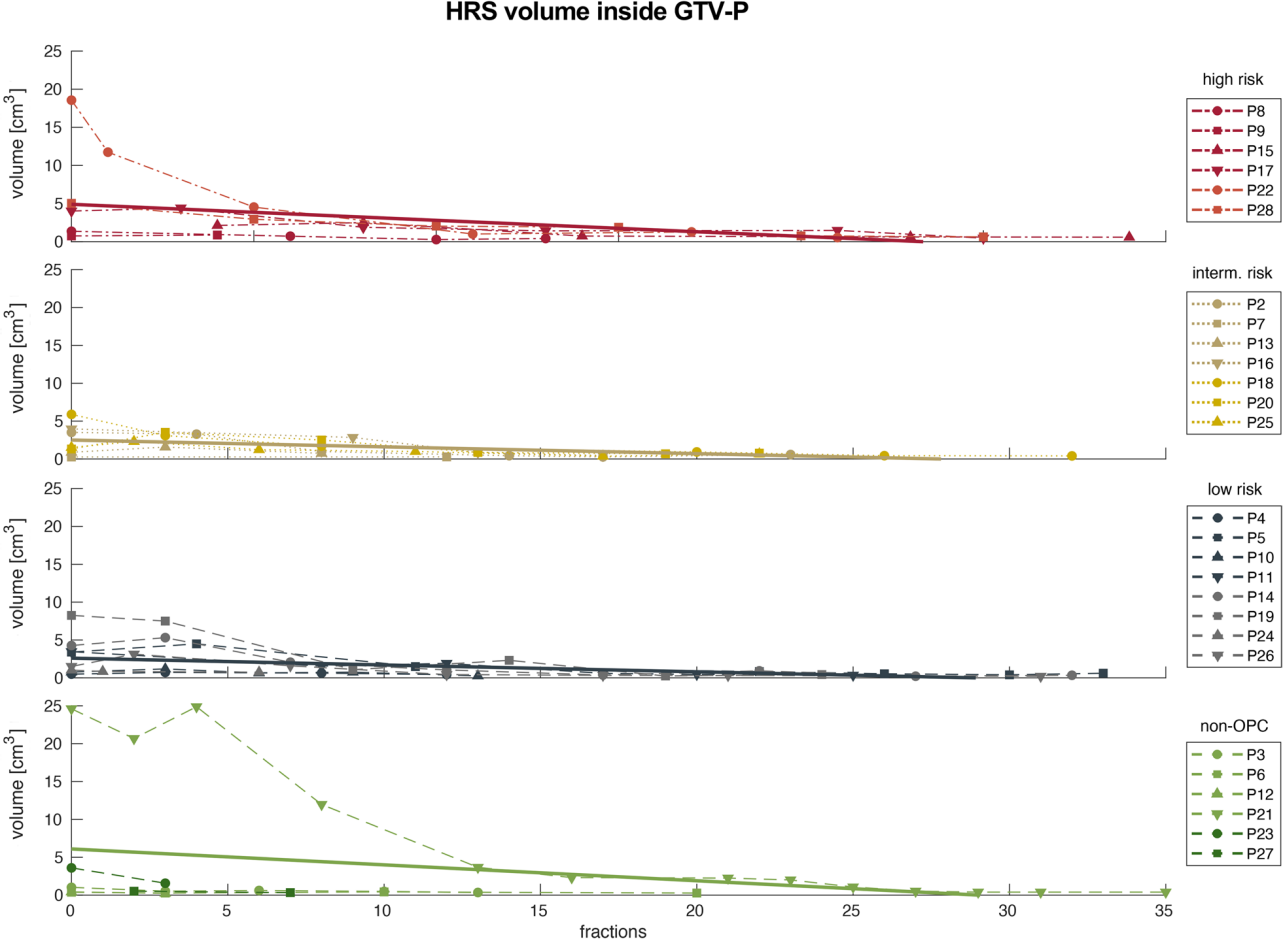
Trend for HRSs inside GTV-P over the course of radiotherapy for high (red), intermediate (gold), low risk (blue) and non-oropharyngeal (green) patients. Solid lines represent linear regression for each group of patients

**Fig. 7:**
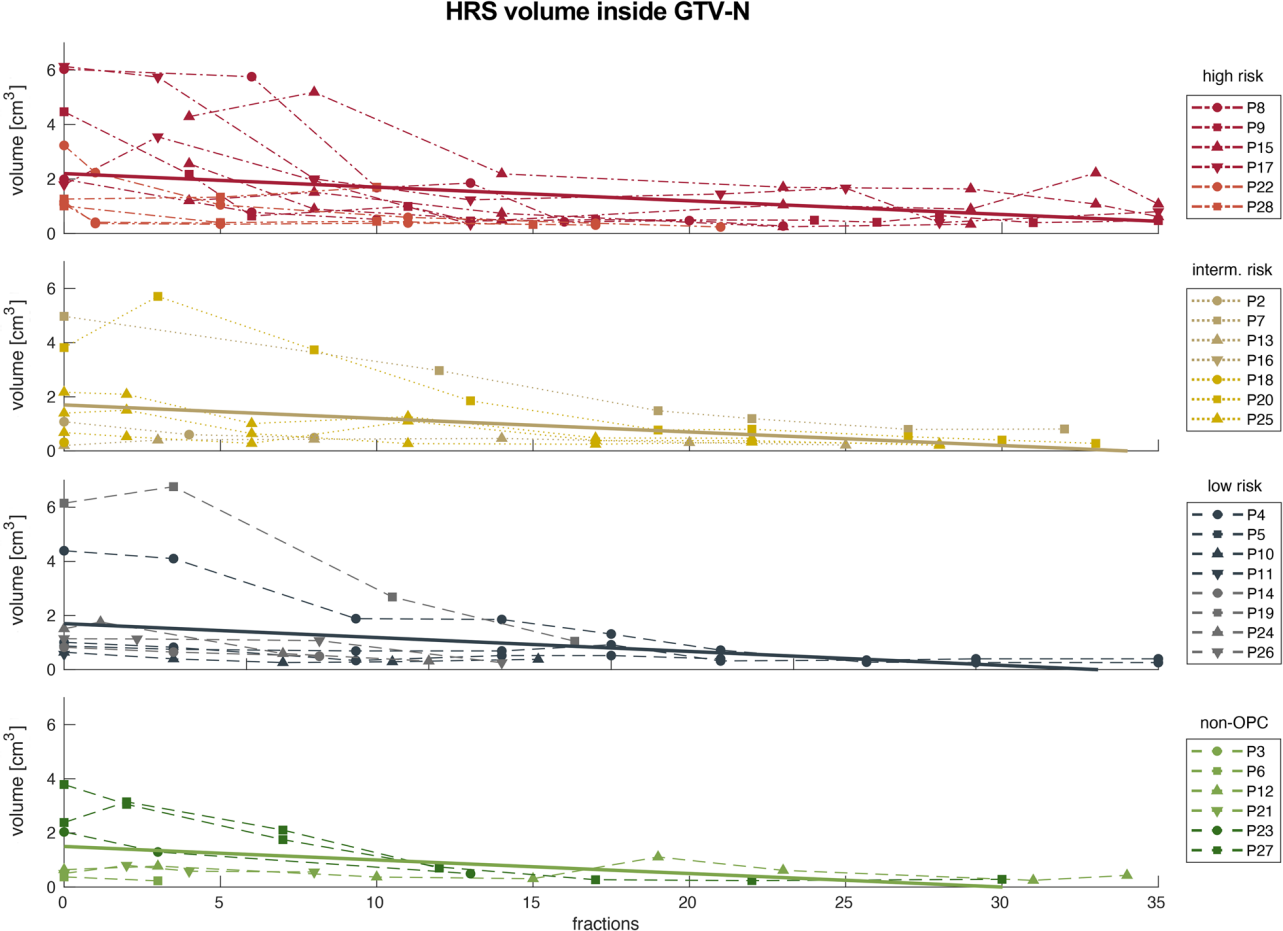
Trend for HRSs inside each individual GTV-N over the course of radiotherapy for high (red), intermediate (gold), low risk (blue) and non-oropharyngeal (green) patients. Solid lines represent linear regression for each group of patients

**Fig. 8:**
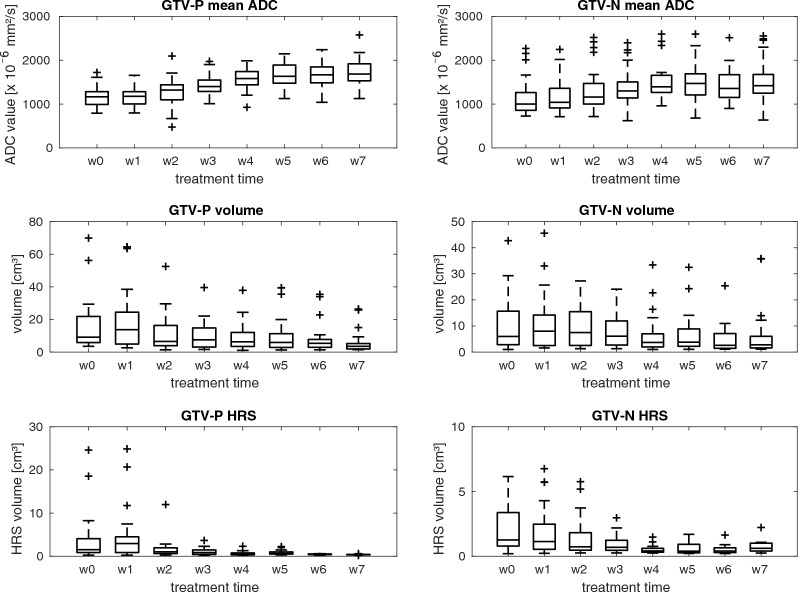
Boxplots of mean ADC, absolute tumor volumes and absolute HRS volumes for all patients before and in every week of radiotherapy

**Fig. 9:**
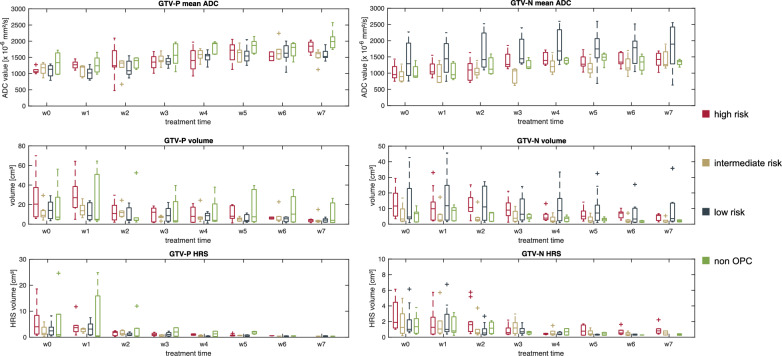
Boxplots of mean ADC values (top) and absolute tumor volumes (bottom) pretreatment (w0) and during radiotherapy (w1–w7) for high risk (red), intermediate risk (gold), low risk (blue) and non-oropharyngeal (green) patients

The ADC-based HRS of the GTV-P and GTV-N showed a median pretreatment size of 1.5 (3.1) cm^3^ and 1.3 (2.4) cm^3^, respectively. In the last week of RT, the median HRS had shrunk to 0.4 (0.0) cm^3^ for GTV-P and 0.6 (0.5) cm^3^ for GTV-N. Figures 6 and 7 show the trend of HRS for all analyzed patients and the linear regression. Detailed information about ADC_mean_, absolute tumor volume and HRS volumes for every risk group pretreatment and in the last week of RT are presented in Table 3. Additionally, absolute values for ADC_mean_, tumor volume and HRS are summarized on a weekly basis for all patients in Fig. 8 and separated by risk groups in Fig. 9.

**Table 3:** ADC_mean_, absolute tumor volumes and HRS volumes before the start of radiotherapy (RT), in week 7 of RT and the relative change over course of RT separated by risk groups

Median (interquartile range) ADC_mean_ [× 10^−6^ mm^2^/s]	High risk		Intermediate risk		Low risk		Non-oropharyngeal	
	GTV-P	GTV-N	GTV-P	GTV-N	GTV-P	GTV-N	GTV-P	GTV-N
Pretreatment	1057 (152)	952 (358)	1181 (350)	890 (309)	1134 (354)	1292 (1112)	1339 (687)	913 (364)
Week 7 of RT	1845 (386)	1420 (410)	1615 (184)	1264 (537)	1532 (198)	1895 (1211)	1987 (416)	1352 (184)
**Median (interquartile range) volumes (cm**^**3**^**)**								
Pretreatment	20.5 (41.4)	11.7 (15.8)	9.1 (7.7)	3.3 (10.6)	14.0 (17.4)	4.6 (20.7)	7.2 (32.4)	6.8 (6.6)
Week 7 of RT	4.0 (3.0)	5.6 (3.9)	2.8 (1.6)	1.5 (2.2)	4.2 (3.8)	3.6 (12.1)	4.0 (24.2)	2.1 (1.3)
**Median (interquartile range) HRS (cm**^**3**^**)**								
Pretreatment	4.0 (10.7)	1.9 (3.7)	1.5 (3.1)	1.3 (3.0)	2.5 (3.3)	1.0 (2.3)	1.0 (13.7)	1.3 (2.3)
Week 7 of RT^a^	–	0.8 (0.6)	0.4	0.3, 0.8	0.2, 0.3, 0.6	–	0.4, 0.4	0.2, 0.4

^a^In case of three or less data points, all datapoints are presented

**Fig. 10:**
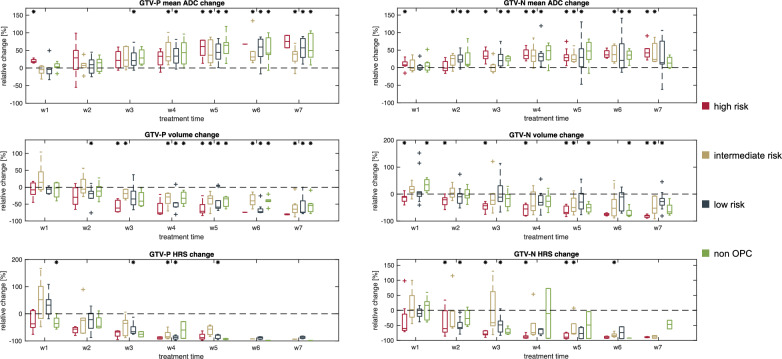
Boxplots of relative change of mean ADC values (top) and tumor volumes (bottom) from baseline to every week of radiotherapy (w1–w7) for high risk (red), intermediate risk (gold), low risk (blue) and non-oropharyngeal (green) patients. Outliers are shown with a plus sign. Significant different changes (*p* < 0.05) from 0 are indicated by a star

Median ADC_mean_ increase to the last week of radiotherapy was 49 (52)% for GTV-P and 24 (48)% and GTV-N. GTV-P volumes showed a median decrease of 68 (24)% while GTV-N volumes decreased by 52 (62)% to the last week of RT. Furthermore, median decrease of HRS volumes yielded 93 (9)% for GTV-P and 87 (18)% for GTV-N. Significant differences (*p* < 0.05) for relative change of ADC_mean_ of GTV-P and GTV-N were found starting in week 1 of RT while tumor volumes showed significant differences (*p* < 0.05) beginning in week 2 for GTV-P and week 1 for GTV-N, respectively. HRS size displayed significant differences (*p* < 0.05) between baseline and week 1 of RT for GTV-P and week 2 for GTV-N. Furthermore, we evaluated the relative changes of ADC_mean_, absolute tumor volumes and HRS size depending on patient risk stratification from baseline to every week of radiotherapy (cf. Figure 10). All clinical risk categories showed an increase of ADC parameters during treatment and decrease in HRS during treatment.

## Discussion

As new QIBs need to undergo technical and also clinical validation with respect to different features such as reliability and repeatability, the aim of the present analysis was to show clinical feasibility of online acquired, longitudinal DWI on a MR-Linac during fractionated RT for HNC and to characterize ADC dynamics. Previously, we reported relative repeatability coefficients (relRCs) of DWI in HNC on the MR-Linac to be 31% for GTV-P and 23% for GTV-N [14] and performed a comparison with a diagnostic scanner as a benchmark [13]. In this study, we observed ADC_mean_ changes larger than these relRCs in weeks 3–4 for GTV-P and weeks 2–3 for GTV-N which correspond to the timepoints where significant ADC changes from 0 could be measured. Hence, week 3 seems to be a reasonable timepoint for ADC based interventions on a MR-Linac.

The current patient cohort is one of the largest to date reporting longitudinal ADC during RT and one of the first to report on online acquired serial DWI. As anticipated and in congruence with previously published data, GTV-P and GTV-N volume decreased during treatment [22, 32, 33]. El-Habashy et al. showed a decrease of GTV-P and GTV-N volumes during fractionated RT for 30 patients treated on an 1.5 T MR-Linac [34]. These volume changes built the rationale for prospective adaptive RT trials such as the MR-ADAPTOR [35, 36]. Our results also show a consistent increase in mean ADC in GTV-P and GTV-N, as reported by others in an offline setting, too [12, 15, 17, 22].

One major limitation is the inherent high technical uncertainty related to the acquisition and analysis of DWI. While echo-planar-imaging (EPI) for DWI has the advantage of fast image acquisition, it severely suffers from geometrical distortions caused by inhomogeneities of the magnetic field [37–39]. EPI applications in HNC patients are especially prone to geometric distortions because of many air-tissue boundaries in the respective area which cause magnetic field inhomogeneity and therefore geometric distortions. Improvements could be achieved by using image acquisition techniques like turbo-spin-echo (TSE) or split acquisition of fast spin echo signal (SPLICE), but with the downside of lower signal-to-noise ratio (SNR) and longer acquisition times [30, 38, 40]. Furthermore, comparability of ADC values and the translation of results from other studies is complicated due to inconsistencies in selected b-values for the DWI sequences and different algorithms used for ADC calculation, different magnetic field strengths and imaging techniques which all might affect the reported ADC values [30, 41, 42]. Another limitation is the small number of patients receiving their treatment within a planned period of time, limiting the potential prognostic value of the analysis as discussed above. Therefore, the MR-Linac consortium is working on publishing consensus DWI sequences or guidelines for the 1.5 T MR-Linac to form a prospective large group of patients, scanned with a dedicated DWI sequence and to make the translation and interpretation of ADC values from different studies easier and to shed further light on the role of DWI and its prognostic value for HNC.

In a preclinical trial, tumour subvolumes based on a cluster of ADC values were found to be significantly associated with radiation sensitivity and local tumor control [26]. In this clinical cohort this association was not reproduced, as the number of events was very small and moreover the reproducibility for serial DWI measurements might be worse than in a classical diagnostic setup and especially compared to a preclinical setting. In contrast to other reported data [15], we could not find a difference between OPC and non OPC or between HPV associated OPC and non-HPV associated OPC, possibly due to the small subgroups.

With no isolated local or regional recurrence, likely due to sampling bias, no correlation of DWI with outcome in this small cohort could be generated. Moreover, the established clinical risk groups for OPC could not be differentiated by the means of mean ADC or ADC-based HRS, potentially due to the small number of patients in each subgroup. Although no obvious differences could be seen between the subgroups in OPC or non-OPC, the high risk subgroup had larger tumor volumes and showed initially a higher HRS, consistent with the potential as a QIB, whereas intermediate and low risk subgroups had already a median HRS below the proposed threshold of 5.8 cm^3^ [27]. The missing correlation with outcome seen in the present analysis, emphasizes the need of larger cohorts of patients and a group of patients suffering from less selection bias. In addition, the HRS even in the high risk and the non-OPC subgroup decreased very rapidly and were below the established threshold of 5.8 cm^3^ already in week 2 for all but one of the patients (Fig. 6). This, together with the significant decrease in various parameters already early during treatment (Fig. 10) emphazises the need of repeated imaging to access the dynamic change of imaging based biomarkers and needs to be taken into account for future QIB-based clinical trials. Moreover, tumor volume in some cases was very small and the resolution of the DWI sequence may affect the analysis especially in the ADC-based HRS at late time points during RT. The seen GTV volume and ADC changes early during treatment offer a further explanation for the so far inconclusive and conflicting body of literature on the potential role of DWI as a prognostic or even predictive marker in HNC [15, 16, 18, 27, 43–45]. Nevertheless, the potential of repeated online quantitative imaging and anlysis of ADC-based subvolumes may facilitate the possibility for an ADC-based dose individualization and emphasizes the consideration of adaptive approaches during QIB-based trials.

## Conclusions

In conclusion, this study shows the feasibility of online acquired, longitudinal DWI on a tumor level as well as in ADC-based subvolumes during the course of MR-guided RT for HNC, which might build the basis of ADC-based biological individualized online adaptive RT trials. The validation of these results in a prospective multicenter study seems to be an important next step.

## Data Availability

The datasets generated and analysed during the current study are not publicly available due to inclusion in an ongoing prospective registry for secondary multi-center analysis but are available from the corresponding author on reasonable request.

## Abbreviations

ADC: Apparent diffusion coefficient
CT: Computed tomography
DCE: Dynamic contrast enhanced
DWI: Diffusion-weighted imaging
EPI: Echo-planar imaging
FDG: Fluorodeoxyglucose
GTV-N: Conspicious lymph nodes
GTV-P: Primary tumor
HNC: Head and neck cancer
HPV: Human papilloma virus 16
HRS: High-risk subvolume
MRI: Magnetic resonance imaging
MR-Linac: Magnetic resonance linear accelerator
OPC: Oropharyngeal cancer
PET: Positron emission tomography
QIB: Quantitative imaging biomarker
relRC: Relative repeatability coefficient
RT: Radiotherapy
SNR: Signal-to-noise ratio
SPLICE: Split acquisition of fast spin echo signal
SS-EPI: Single-shot echo-planar imaging
TSE: Turbo-spin-echo
VOI: Volume of interest
